# Supplementary material for: Biostimulant and Bioinsecticidal Effect of Coating Cotton Seeds with Endophytic Beauveria bassiana in Semi-Field Conditions
Source: Microorganisms. 2023 Aug 9;11(8):2050. doi: 10.3390/microorganisms11082050 (PMC10457994; doi:10.3390/microorganisms11082050)
Supplement: Supplementary file 1 [file microorganisms-11-02050-s001.zip › microorganisms-2525530-supplementary.pdf]

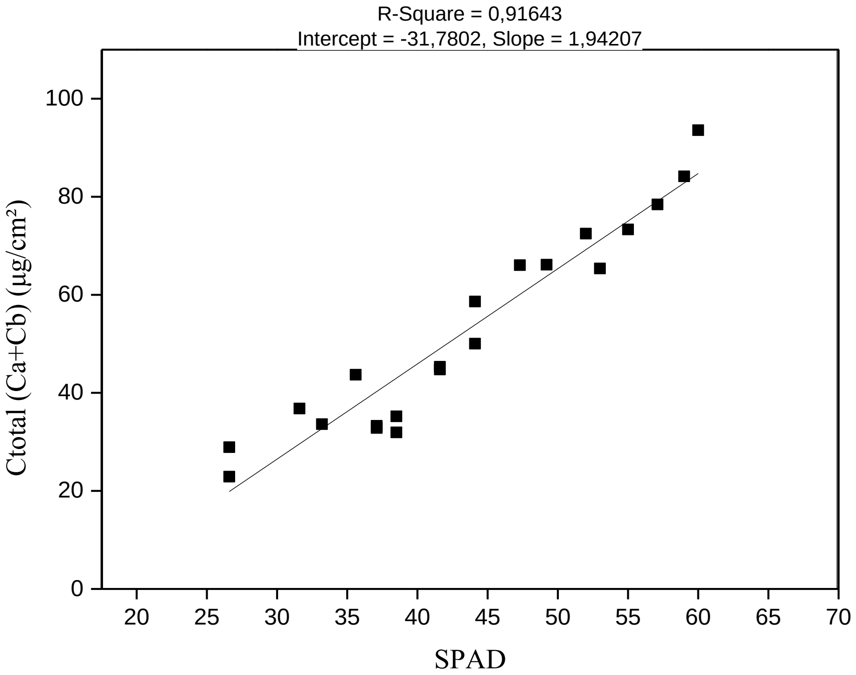

Figure S1. Correlation between SPAD readings and total chlorophyl concentration in cotton leaves.
